# Natural Sesquiterpene Lactones Enhance Chemosensitivity of Tumor Cells through Redox Regulation of STAT3 Signaling

**DOI:** 10.1155/2019/4568964

**Published:** 2019-10-28

**Authors:** Elena Butturini, Alessandra Carcereri de Prati, Diana Boriero, Sofia Mariotto

**Affiliations:** Department of Neuroscience, Biomedicine and Movement Sciences, Section of Biological Chemistry, University of Verona, Strada Le Grazie, 8, 37134 Verona, Italy

## Abstract

STAT3 is a nuclear transcription factor that regulates genes involved in cell cycle, cell survival, and immune response. Although STAT3 activation drives cells to physiological response, its deregulation is often associated with the development and progression of many solid and hematological tumors as well as with drug resistance. STAT3 is a redox-sensitive protein, and its activation state is related to intracellular GSH levels. Under oxidative conditions, STAT3 activity is regulated by S-glutathionylation, a reversible posttranslational modification of cysteine residues. Compounds able to suppress STAT3 activation and, on the other hand, to modulate intracellular redox homeostasis may potentially improve cancer treatment outcome. Nowadays, about 35% of commercial drugs are natural compounds that derive from plant extracts used in phytotherapy and traditional medicine. Sesquiterpene lactones are an interesting chemical group of plant-derived compounds often employed in traditional medicine against inflammation and cancer. This review focuses on sesquiterpene lactones able to downmodulate STAT3 signaling leading to an antitumor effect and correlates the anti-STAT3 activity with their ability to decrease GSH levels in cancer cells. These properties make them lead compounds for the development of a new therapeutic strategy for cancer treatment.

## 1. Introduction

Cancer is the main single cause of death in both men and women, claiming over 6 million lives each year worldwide. The hallmarks of cancer include tumor cell proliferation and survival, tumor angiogenesis, and metastasis. Tumor cells exhibit an altered metabolism that allows them to sustain high proliferative rates and resist to some cell death signals, particularly those mediated by increased oxidative stress. Several studies have identified a critical role of aberrant activation of STAT3 signaling in oncogenesis. Therefore, any treatment counteracting the STAT3 hyperactivation has been considered as a new strategy to treat different tumors.

Over the last 20 years, a lot of literature evidence indicates that many derived plant substances are potentially interesting in cancer therapy or can be considered as lead compounds to develop new possible anticancer drugs.

## 2. Signal Transducer and Activator of Transcription 3

### 2.1. STAT3 Structure

Signal transducer and activator of transcription 3 (STAT3) is a member of a family of seven proteins (STAT 1, 2, 3, 4, 5a, 5b, and 6) activated by growth factors and cytokines that participate in physiological cellular responses [1, 2]. The transcript of STAT3 undergoes alternative splicing, resulting in the full length STAT3*α* (92 kDa) and in the truncated isoform STAT3*β* (83 kDa) that lacks the C-terminal domain including Ser727 [3].

Two crystal structures of STAT3 are deposited in the Protein Data Bank (PDB): the phosphorylated STAT3*β*-DNA complex (1BG1) [4] and the unphosphorylated STAT3 core fragment (3CWG) [5]. Sequence comparisons, biochemical assays, and mutagenesis have identified six functional conserved domains within the STAT3 molecule, each of them contributing to various aspects of signal transduction pathway. The domains are arranged in the protein structure as follows: an N-terminal domain (NTD) (1-137), a coiled-coil domain (CCD) (138-320) formed by a four-helix bundle, a DNA-binding domain (DBD) (321-494) comprising an eight-stranded *β*-barrel, a *α*-helical linker domain (LD) (495-583), a Src homology 2 (SH2) domain (584-688), and a C-terminal transcriptional activation domain (TAD) (723-770). The NTD is a conserved sequence that mediates tetramerization of two phosphorylated dimers which cooperatively bind specific STAT3 sites in a gene promoter [6, 7]. The CCD is critical for recruitment of STAT3 to the receptor, subsequent phosphorylation and dimerization, and its translocation into the nucleus [8]. Moreover, the CCD is involved in protein-protein interactions leading to multiple types of dimer complexes, and it also contains a lysine residue (Lys140) subject to methylation by histone methyl transferase SET9, which is a negative regulatory event [9]. The DBD allows the recognition and the binding to a specific consensus sequence defining the DNA-binding specificity. The SH2 domain is required for the recruitment of signal transduction proteins to activated receptors and contains a key binding pocket where the phosphotyrosine residue of other STAT proteins can bind to form homo- or heterodimers [10]. Other than SH2 domain interaction, we have recently detected two interchain disulfides between cysteine 367 and cysteine 542 and between cysteine 418 and cysteine 426 (Cys367-Cys542 and Cys418-Cys426) responsible for STAT3 dimer stabilization [11]. Finally, the TAD is involved in transcriptional activation and promotes the full STAT3 activation through the phosphorylation of the serine residue 727 (Ser727). In the C-terminal domain, between SH2 and TAD, there is a tail segment with the phosphorylation site tyrosine 705 (Tyr705) that controls dimerization and yields the DNA-binding activity of STAT3 [12].

### 2.2. STAT3 Signaling Cascade

**Figure 1:**
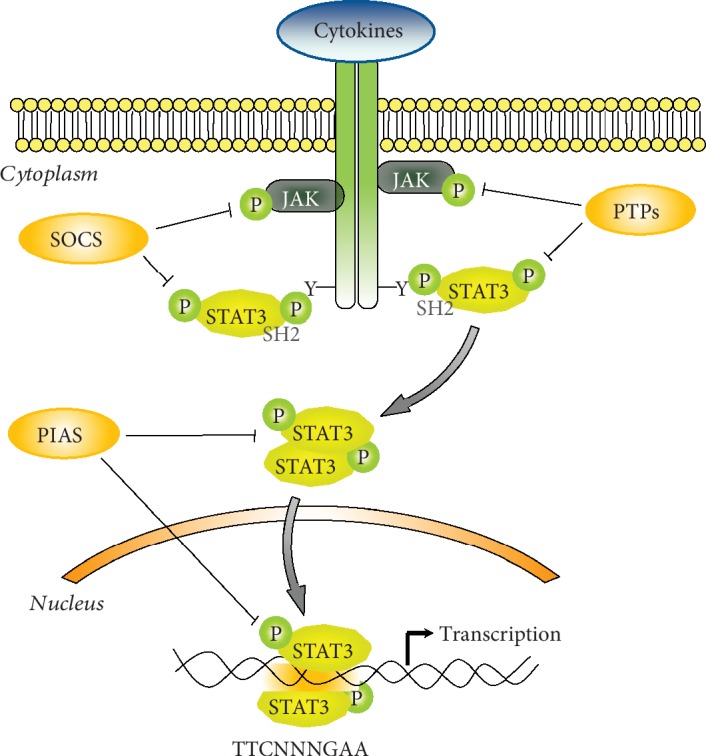
STAT3 signaling pathway. p-STAT3: phosphorylated STAT3; p-JAKs: phosphorylated JAKs; SOCS: suppressor of cytokine signaling proteins; PIAS: protein inhibitors of activated STATs; PTPs: protein tyrosine phosphatases.

Multiple distinct steps are involved within the STAT3 signaling pathway. According to the classical model, STAT3 is activated through the binding of growth factors and cytokines to their cell-surface receptors. Cytokines, like IL-6, IL-10, and IL-11, as well as growth factors, like endothelial growth factor (EGF), vascular endothelial growth factor (VEGF), and fibroblast growth factor (FGF), can activate the phosphorylation cascade. This event allows rapid transphosphorylation and activation of Janus tyrosine kinases (JAKs, JAK1, JAK2, JAK3, and Tyk2) that phosphorylate tyrosine residues on the cytoplasmic tail of the receptors. The SH2 domain of STAT3 recognizes and binds to these docking sites, placing STAT3 within close proximity of active JAKs, which subsequently phosphorylate STAT3 at Tyr705. The phosphorylated form of STAT3 homo- or heterodimerizes via reciprocal SH2 domain interaction and translocates from the cytoplasm to the nucleus, where it regulates the transcription of target genes (Figure 1) [13, 14]. In addition to JAKs, STAT3 can be activated by nonreceptor tyrosine kinases such as Src and ABL [15–17]. Furthermore, various serine kinases, like protein kinase C (PKC), mitogen-activated protein kinases (MAPK), and CDK5, phosphorylate the OH residue of Ser727. Although serine phosphorylation occurs in several cells, its biologic role is still controversial. Some authors report that serine phosphorylation allows to achieve maximal transcriptional activity [18], whereas others demonstrate that serine phosphorylation inhibits STAT3 activity [19, 20].

The binding of STAT3 to a specific DNA domain promotes the expression of numerous genes involved in cell cycle progression, apoptosis, tumor angiogenesis, invasion, metastasis, chemoresistance, immunosuppression, and cancer stem cell renewal (Table 1) [21–40]. Intriguingly, many downstream target genes of STAT3 encode cytokines and growth factors that trigger the same STAT3 signaling pathway, thereby providing a mechanism of autocrine and paracrine STAT3 activation.

Under physiological conditions, the activation of STAT3 signaling is a transient and tightly regulated process that can last from half an hour to several hours. After this period, the signal decays and STAT3 are exported back to the cytoplasm. This decay entails downregulation of both receptors and JAKs, as well as of STAT3 transcriptional activity, and involves several negative protein modulators, including the family of suppressor of cytokine signaling proteins (SOCS), the protein inhibitors of activated STATs (PIAS), and several protein tyrosine phosphatases (PTPs) [41, 42] (Figure 1).

The SOCS family is composed by eight inducible intracellular proteins, all characterized by the SH2 domain that interacts with phosphorylated JAKs and/or with the intracellular domains of the receptors to impede the recruitment of STATs to the docking sites as well as to inhibit JAK activity. Moreover, via their SOCS box domain, SOCS interact with E3 ubiquitin ligase and promote the ubiquitin-dependent degradation of targets [43]. Specifically, STAT3 stimulates SOCS3 gene transcription and the resulting protein binds phospho-JAKs and/or the receptors to turn off the cascade.

Other than SOCS, STAT3 transcriptional activity is controlled by PIAS3, a nuclear protein member of PIAS family proteins which prevents active STAT3 from binding DNA and inhibits STAT3-mediated gene activation [44].

Furthermore, STAT3 transcriptional activity is controlled by PTPs, a family of tyrosine phosphatases, that operate on various steps of signaling cascade. The best characterization of these proteins is SHP-1 and SHP-2 that contain SH2 domain and ensure that tyrosine phosphorylation of JAKs does not persist after the removal of the cytokine [2, 12]. Inactivation of STAT3 in the nucleus occurs through the dephosphorylation of Tyr705 by TC-PTP and TC45 [45].

There is a growing body of evidence demonstrating that STAT3 signaling is also regulated via a complex interplay with cellular miRNAs. Both direct and indirect regulatory mechanisms mediate several positive and negative feedback loops between miRNAs and the STAT3 signaling pathway. Approximately, 50 miRNAs are predicted to bind the 3′-UTR of STAT3; among them, let-7, miR-20a, and miR-93 were directly validated using STAT3-3′-UTR-Reporter constructs. Several miRNAs directly induce STAT3 upregulation (miR-551b 3p) or act to reduce the expression of negative regulators of STAT3 (miR-18a, miR-221, and miR-222), and others are activated by STAT3 (miR-21) through binding within the promoters of these oncomiRs. A more thorough review can be found in the manuscript by [46].

### 2.3. STAT3 and Oncogenesis

Growing evidence over the last years suggests a critical role of STAT3 as a point of convergence of various signaling pathways that are deregulated in cancer. In healthy cells, STAT3 is closely regulated to maintain a transient active state. Conversely, STAT3 is improperly and persistently activated in numerous hematopoietic and solid malignancies [47, 48]. Constitutively active STAT3 induces deregulation of growth and survival, promotion of angiogenesis, and suppression of host's immune surveillance against tumor. Moreover, it promotes epithelial-mesenchymal transition, invasion, and metastasis thereby contributing to tumor progression. In the last years, increasing evidence indicates that STAT3 also promotes resistance to conventional chemo- and radiation therapy as well as to pharmacological inhibition of several pathways of oncogene-driven malignancies [49, 50].

Although recent studies have revealed activating STAT3 mutations in some malignancies (hepatocellular adenoma, 40% of large granular lymphocytic leukemia, and 30% of chronic lymphoproliferative disease of NK cells), these mutations are too rare to account for the high prevalence of STAT3 activation in solid tumors.

The constitutive activation of STAT3 in cancer is caused mostly by the higher secretion of cytokines and growth factors in tumor microenvironment. Furthermore, in this context, it has been recognized a critical role to the deregulation of receptors with intrinsic tyrosine kinase activity (e.g., EGFR or HER-2/neu) or of nonreceptor tyrosine kinases (e.g., Src or Abl), as well as to the epigenetic modulation of negative regulators of STAT3. High levels of IL-6 have been reported in a lot of cancer patients and are also described as a potent negative regulator of dendritic cell maturation *in vivo*, contributing to control T cell-mediated immune responses [51].

Studies of myeloma, hepatocellular carcinomas, and non-small-cell lung cancer report the loss of proteins that negatively regulate STAT3, such as PIAS [52] or SOCS [53].

On the other hand, JAK mutations and their relevance in the pathogenesis of hematological disorders are well described, with JAK2 V617F being the most well-known mutation, which is found in >95% of patients with polycythaemia vera, primary myelofibrosis, and essential thrombocytosis [54]. Mutations in the genes encoding JAK enzymes seem to be much less common in solid tumors.

Abnormal STAT3 signaling is also associated with defects in activation of JAKs due to a chromosomal translocation resulting in a fusion protein that contains the kinase domain of JAK2 fused to the oligomerization domain of the Ets transcription factor (Tel-JAK2) and possesses constitutive tyrosine kinase activity [55].

It has been reported that also noncanonical pathways of STAT3 signaling play a significant role in malignant transformation, causing alternative posttranslational modifications like phosphorylation of Ser727 and acetylation of Lys685 [56–59].

In the last years, miRNAs are emerging as important regulators of the JAK-STAT3 pathway in the pathogenesis of cancer, causing up- or downmodulation of STAT3 signaling, as well as in the development of chemoresistance in several types of cancer. Further insights on the subject are by [46].

### 2.4. Treatment Strategies Targeting STAT3 Protein

**Figure 2:**
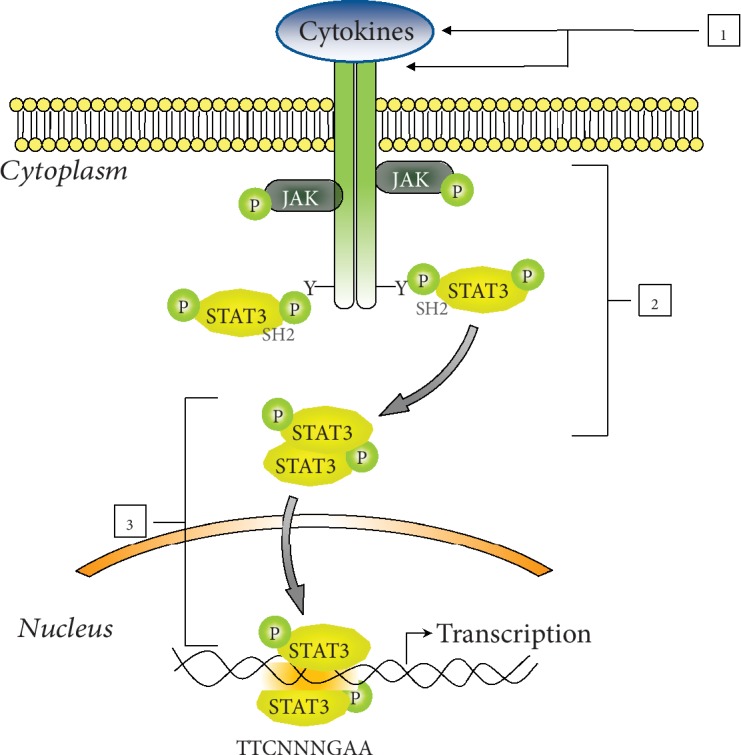
Strategies for inhibition of the STAT3 signaling pathway. Several agents targeting various nodes of STAT3 cascade have been developed. Agents that act on nodes 1 and 2 indirectly switch off STAT3 signaling. Compounds at node 3 directly target STAT3 protein or its DNA-binding downmodulating STAT3 activation. p-STAT3: phosphorylated STAT3; p-JAKs: phosphorylated JAKs.

The understanding that STAT3 signaling promotes tumorigenesis and chemoresistance while severely hinders antitumor immunity has stimulated the search for clinical agents that can effectively inhibit this pathway. Over the last 15 years, many direct or indirect inhibitors targeting various members of the STAT3 pathway have been employed to disrupt STAT3 activity (Figure 2) and some of them entered in clinical trials for treatment of solid or hematological tumor.

Two principal approaches that indirectly inhibit STAT3 activation have been developed. First of all, antibodies that target IL-6 or its receptor are extensively evaluated preclinically and clinically (Figure 2, node 1). Siltuximab and tocilizumab are two antibodies approved by the FDA for the treatment of arthritis or Castleman disease that have been testing in phase I/II clinical trials in different hematological as well as solid tumor [60–65]. Another indirect but efficient mechanism is the use of JAK or Src inhibitors (Figure 2, node 2) [66]. A number of small JAK and Src inhibitors are now in various stages of clinical trials, and some of them result in approved drugs, specifically ruxolitinib and tofacitinib [67]. Other JAK and Src inhibitors such as AZD1480, WP-1066, desatinib, and saracatinib demonstrate the reduction of STAT3 phosphorylation as well as downstream implications like increased apoptosis and decreased tumor growth [68–71]. Unfortunately, the JAK and IL-6 inhibitors determine an increased rate of infection and off-target neurotoxicity. Moreover, the inhibition of these kinases may influence different signaling cascades and give rise to additional off-target effects. For example, the crucial role of STAT1 in inflammatory response and in disrupting cell proliferation is well known, as well as in antitumor and immune surveillance [72–75]. Therefore, STAT1 should not be downregulated, while attempting to inhibit the actions of STAT3. It is clear that further investigation of all these inhibitors is necessary to understand how to optimize STAT3 inhibition.

For all these reasons, a better strategy for STAT3 inhibition is through the direct targeting of functional phosphorylated STAT3. A lot of peptides and small molecules that impair dimerization, nuclear translocation, and DNA binding of STAT3 have been developed (Figure 2, node 3).

The small peptides designed on STAT3 SH2 domain sequence that contain a tyrosine-phosphorylation site (PY^∗^LKTK) bind to the SH2 domain of STAT3 preventing its dimerization and translocation into the nucleus [76, 77]. Although these compounds have proapoptotic and antitumor activity in cancer cells, they have primarily been used as research tools due to their limited cellular uptake and stability.

Nonpeptidic small molecules able to permeate cells represent a more attractive approach to inhibit aberrant STAT3 activity in cancer cells [78]. Compounds, such as STATTIC, STA-21, LLL-3, LLL-12, WP1066, S3I-201, BP-1-102, STX-0119, and HJC0123, inhibit the growth of tumor cells with hyperactivated STAT3 [79–82]. Although many SH2 domain inhibitors have proved to be promising in laboratory studies, only a few have been evaluated in clinical trials.

An alternative approach useful to inhibit STAT3 function involves competitive inhibition of the interactions between DBD domain of STAT3 and promoter elements in target genes. Platinum (IV) complex, such as CPA-1, CPA-7, and IS3-295, inhibits the STAT3 DNA-binding activity leading to apoptosis in human cancer cell lines [83]. A 15 bp double-stranded decoy oligonucleotide that correspond to the STAT3 response element in the c*FOS* promoter competitively inhibits STAT3 DNA binding and suppresses the tumor growth of preclinical models of ovarian, breast, head-and-neck, lung, brain, and skin cancers as well as acute myeloid leukemia [84–87].

Although many of these anti-STAT3 compounds have antitumor effects *in vitro* and *in vivo*, there are no currently approved drug directly targeting STAT3 and the research of STAT3 inhibitors is still evolving.

## 3. Redox Homeostasis in Cancer Cells

### 3.1. Intracellular Redox Homeostasis

In contrast to normal tissue, most of solid tumors are characterized by regions of low oxygen (hypoxia), low pH, and low levels of glucose which result from an architecturally abnormal microcirculation, rapid growth of tumor cells, and high interstitial pressure. Hypoxia and the high energetic metabolism induced by tumor microenvironment contribute to upregulation of reactive oxygen species (ROS) production in mitochondria, peroxisomes, and endoplasmic reticulum [88–91]. Excessive levels of ROS cause oxidative damage to DNA, proteins, and lipids, compromising their structures and function. To prevent oxidative damage, cancer cells activate various enzymatic and nonenzymatic antioxidant systems. The first ones include superoxide dismutase, catalase, glutathione peroxidase, and glutathione reductase whereas *α*-tocopherol (vitamin E), *β*-carotene (vitamin A), ascorbic acid (vitamin C), and uric acid represent the ROS scavenging molecules. Furthermore, multiple and interrelated redox couples, such as NADPH/NADP^+^, GSH/GSSG, Trx/TrxSS, and cysteine/cystine, contribute to the intracellular redox homeostasis [92–99].

A number of human cancer tissues, including breast, brain, colon, pancreas, lungs, and leukemia, produce high concentrations of glutathione (GSH) that contribute to cancer initiation, progression, and metastasis formation and to chemoresistance [100–103]. In accordance with the elevated level of GSH in cancer cells, several drugs known to reduce GSH concentration are currently being used in clinical trials to improve efficacy of targeted therapy. In this regard, the use of disulfiram, alone or combined with arsenic trioxide, has been approved as therapy for metastatic melanoma and nonacute promyelocytic leukemia [101, 104]. Buthionine sulfoximine (BSO), a synthetic inhibitor of GSH production, confers increased sensitivity to chemotherapy in myeloma and neck cancers [105] and has been clinically used in various types of cancers [106]. Similarly, phenylethyl isothiocyanate (PEITC), which conjugates with GSH, inhibits the oncogenic transformation of ovarian epithelial cells and hematopoietic cells [107].

Collectively, modulation of the GSH level is an alternative way to increase the sensitivity of tumor cells to conventional chemotherapy and provides a viable option for patients suffering from therapy-resistant tumors.

### 3.2. [GSH]/[GSSG] Redox Couple

The tripeptide glutathione (Glu-Gly-Cys) is the most abundant intracellular nonenzymatic ROS scavenger reaching millimolar concentrations in the cells. Intracellular glutathione can exist as a monomer in its reduced form (GSH) or as a disulfide dimer (GSSG) after its oxidation which usually accounts for less than 1% of the total intracellular glutathione content.

**Figure 3:**
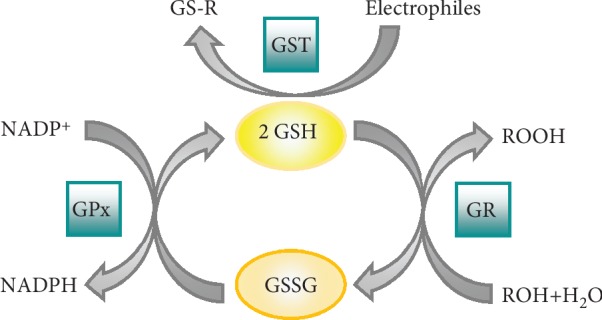
Antioxidant function of glutathione. GST: glutathione transferase; GS-R: electrophile-GSH adduct; GPx: glutathione peroxidase; GR: glutathione reductase.

As antioxidant and intracellular redox buffer, GSH has essential roles in ROS scavenging and in detoxification of electrophiles, xenobiotics, and heavy metals. Two oxidized GSH molecules dimerize by a SS bond to form GSSG. Glutathione reductase, a NADPH-dependent enzyme, reverts this reaction to reconstitute GSH pool. GSH reduces peroxides and generates GSSG via glutathione peroxidase (GPx) or it reacts with many electrophiles to generate glutathione *S*-conjugates (GS-R). Although these reactions can occur spontaneously, they are often catalyzed by the glutathione *S*-transferase (GST) [108, 109] (Figure 3).

The cellular redox status can be evaluated measuring the GSH/GSSG ratio by the Nernst equation [110]. At 25°C and pH 7, *E*° of the GSH/GSSG redox couple is

(1) $$\begin{array}{c} \text{GSSG}+2{\text{e}}^{-}+2{\text{H}}^{+}⇆2\text{GSH}\;{E^{\circ }}_{\text{GSH}/\text{GSSG}}=-240\text{ mV}. \end{array}$$

Since two GSH molecules are needed to form one GSSG molecule, the reaction is second order with respect to GSH. Thus, any changes in the absolute concentration of GSH will change the redox potential, even without changes in the GSH/GSSG ratio. This suggests that cells with much higher GSH level have a greater reducing capacity than cells with lower GSH concentration.

The cellular redox state is one of the master regulators of different cellular processes, and physiological cellular function is maintained by a fine balance between reducing and oxidizing conditions. It has been reported that the etiology and/or progression of many human diseases, including cancer, are related to GSH/GSSG homeostasis. Generally, elevated levels of GSH that determine a more reducing cellular environment stimulate cell proliferation whereas a mild oxidizing environment results in cell differentiation. A further shift toward a more oxidant cellular environment leads to apoptosis or necrosis [108, 110, 111].

### 3.3. Redox Regulation of STAT3

Under oxidative stress, many proteins undergo reversible and irreversible *oxidative* modifications, which may lead to changes in the structure and/or function of the oxidized protein. These redox-sensitive proteins exhibit a striking differential susceptibility to oxidative stress; while a protein may contain numerous residues, only a minority of them will have the chemical properties to function as a possible target site for oxidant. This is largely due to the reactivity of anionic sulfur of various oxidizing agents.

Mild oxidative stress induces selective modifications of proteins at critical cysteine thiols including reversible oxidation to sulfenic acids, intra- and intermolecular disulfides, S-glutathionylation, and S-nitrosylation [112]. S-Glutathionylation, the reversible formation of protein-mixed disulfides with GSH, represents the most common steady-state derivative due to cellular abundance of GSH and ready conversion of cysteine-sulfenic acid and S-nitrosocysteine precursors to S-glutathionylcysteine disulfides. This reaction may protect proteins from irreversible damage or modulate protein function. Conversely, excessive oxidative stress is associated with permanent loss of function, misfolding, and aggregation due to irreversible modification of SH groups of protein [113–115].

Several studies demonstrate that intracellular redox environment influences STAT3 activation cascade although it is still not clear if ROS up- or down-regulate STAT3 activation. Some authors report that ROS trigger Tyr705 STAT3 phosphorylation and upregulate its DNA-binding activity [116, 117]. On the other hand, other authors indicate that ROS oxidize conserved cysteines in STAT3 DNA-binding domain impairing its transcriptional activity [118, 119]. Moreover, there is evidence from the literature which prove that ROS scavengers and inhibitors of NADPH oxidase enzymes (NOX) generally inhibit STAT3 activity [120, 121]. In addition, it has been shown that nitrosocyclohexyl acetate, a nitroxyl donor, inhibits STAT3 phosphorylation through the formation of sulfenic acid at the cysteine residues in endothelial cells [122].

S-Glutathionylation and S-nitrosylation inhibit STAT3 phosphorylation as well as its DNA-binding activity in different cell lines and in *in vitro* studies. Although the 3D model of nitrosylated/glutathionylated STAT3 is not available, it can be speculated that the small conformational changes induced by NO or GSH addition could in turn induce a conformational change in the phosphorylation site of protein inhibiting accessibility to JAKs [119, 123–125].

Our group has been studying STAT3 redox regulation for the past ten years. Particularly, we identified three sesquiterpene lactones, costunolide, dehydrocostuslactone, and cynaropicrin, able to inhibit IL-6-induced as well as constitutive activation of STAT3 in different cancer cell lines. These compounds disrupt intracellular redox homeostasis, induce reversible S-glutathionylation of STAT3, and decrease its Tyr705 phosphorylation [126, 127]. Deepening inside the redox regulation of STAT3 signaling, we reported that Cys328 and Cys542 in the DNA-binding domain and in the linker domain, respectively, are a target of S-glutathionylation [123, 128].

Since STAT3 is validated as a therapeutic target in different solid and hematologic tumor, the modulation of oxidative stress could be a new strategy to inhibit STAT3 hyperactivation. On the other end, the consequent decrease in GSH levels could sensitize tumor cells to conventional chemotherapy.

## 4. Sesquiterpene Lactones

### 4.1. Sesquiterpene Lactone Structure

Sesquiterpene lactones (SLs) are colorless, bitter, and stable compounds of terpenoids, a class of lipophilic plant secondary metabolites. More than 5000 SLs have been characterized in species of the plant kingdom, in particular in the family Asteraceae, and plant extracts rich in SLs have long been employed in traditional medicine against inflammatory-related diseases. SLs possess a broad spectrum of biological activities, including anti-inflammatory, antibacterial, and immunomodulatory effects. These compounds also inhibit cell cycle and proliferation and induce apoptosis, in different cancer cell lines and in many *in vivo* studies [129–131]. Although the exact mechanisms of action are not well elucidated, emerging data suggest that the biological effect of SLs is associated with depletion of GSH and ROS generation [126, 127, 132, 133].

**Figure 4:**
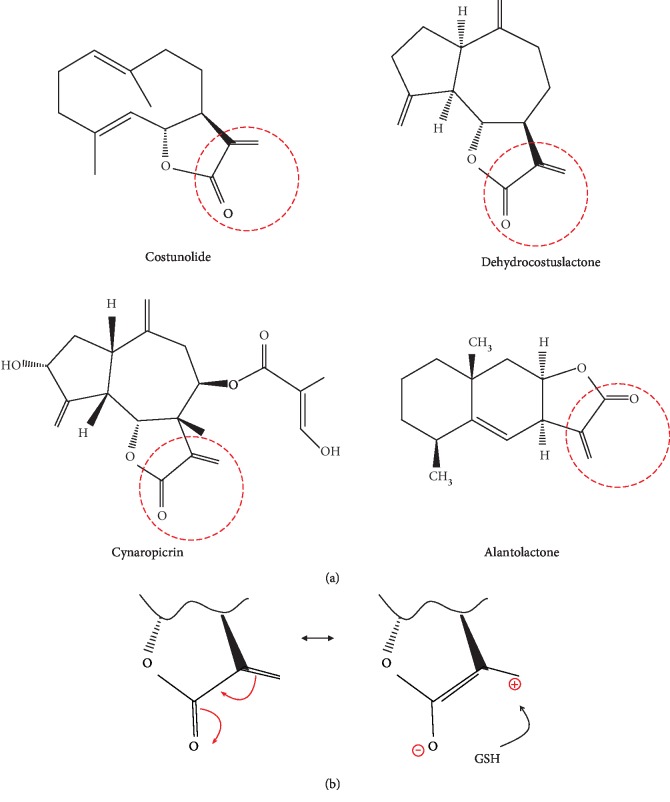
(a) Chemical structure of SLs that induce STAT3 S-glutathionylation and impair STAT3 phosphorylation. The reactive centre of SLs is evidenced with a red circle. (b) Schematic representation of Michael reaction.

SLs are 15 carbon compounds consisting of three isoprene (5-C) units arranged in several characteristic ring systems, including one or more lactone rings. The *α*-*β*-unsaturated carbonyl group present in most of these compounds is the major responsible for their biological effects [134]. The *α*-*β*-unsaturated carbonyl group is a strong alkylating agent that may react by Michael-type addition with intracellular nucleophiles, such as cysteine sulfhydryl residues in proteins, leading to disruption of their biological function. The *α*-*β*-unsaturated carbonyl moiety may also react with the sulfhydryl group of cysteine residue in GSH leading to redox homeostasis disruption and oxidative stress in cells (Figure 4(b)) [134–136].

Further chemical features, such as lipophilicity and molecular geometry of compounds as well as the chemical environment of the target nucleophiles, also influence the bioavailability and biological activity of SLs [137, 138].

### 4.2. Sesquiterpene Lactones and STAT3

In the last years, many natural SLs able to induce apoptosis through the inhibition of STAT3 signaling have been recognized in different cancer cellular and animal models (Table 2) [126, 127, 139–155]. Induction of apoptosis was found to be linked with increased ROS production, GSH depletion, and modulation of GSH/GSSG ratio. Although the final biological outcome of all SLs is well described, the molecular mechanism of anti-STAT3 activity is not reported for all of them. Cheng et al. demonstrate that 6-O-angeloylplenolin directly interacts with the SH2 domain of STAT3 and inhibits the constitutive and IL-6-induced STAT3 activity in lung cancer cells [153]. A direct interaction with STAT3 SH2 domain is also reported for alantolactone [142]. Furthermore, Liu et al. describe that parthenolide covalently binds to Cys residues of JAKs suppressing its kinase activity and downmodulating the STAT3 pathway [145].

Other studies show that SLs inhibit STAT3 signaling through S-glutathionylation of Cys residues in STAT3 protein. Dehydrocostuslactone, costunolide, cynaropicrin, and alantolactone that contain an *α*-*β*-unsaturated carbonyl group directly interact with GSH by Micheal addition and induce a rapid drop in GSH concentration, thereby triggering S-glutathionylation of STAT3. This event impairs STAT3 phosphorylation switching off the signaling cascade (Figure 4(b)) [126, 127, 139]. It is possible to speculate that S-glutathionylation is the common molecular mechanism of anti-STAT3 activity of other SLs able to disrupt GSH/GSSG homeostasis [140, 141, 143, 146–148, 150–152]. The exact molecular mechanism by which S-glutathionylation inhibits STAT3 phosphorylation is not completely clarified. We reported that S-glutathionylation of STAT3 slightly modulates the secondary and tertiary structure of STAT3 affecting the phosphorylation site thus hampering the recognition of Tyr705 site by JAKs [123].

Various *in vitro* and *in vivo* studies reveal that suppression of STAT3 activation by SLs overcomes drug resistance [127, 139, 142, 144, 152, 154, 155]. Since the central role of STAT3 in carcinogenesis and chemoresistance, SLs able to switch off STAT3 signaling have gained considerable attention from the researchers for the development of a new therapeutic strategy for cancer treatment.

## 5. Concluding Remarks

**Figure 5:**
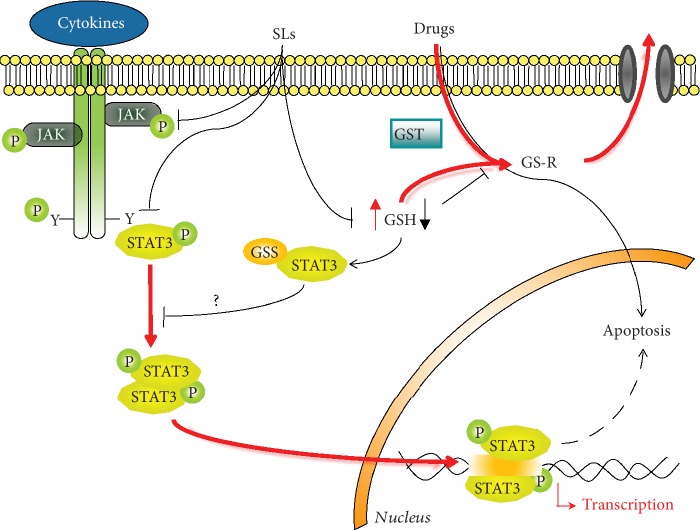
Cancer cells are characterized by elevated levels of GSH that confer resistance to several chemotherapeutic drugs and by constitutive activation of STAT3 signaling that contributes to tumorigenesis and tumor growth, promotes angiogenesis and metastasis, suppresses immune response, and induces chemoresistance (red line). SLs inhibit STAT3 signaling targeting different steps in the signaling cascade (black line). The mild oxidative stress, derived by the direct binding of SLs to GSH, induced S-glutathionylation of STAT3 switching off STAT3 signaling (black line). Moreover, the reduced GSH levels contribute to overcome chemoresistance. GST: glutathione transferase; GS-R: drug-GSH adduct; GSS-STAT3: glutathionylated STAT3; p-STAT3: phosphorylated STAT3; pJAKs: phosphorylated JAKs.

Very often, the rational development of drugs that kill cancer cells interacting with one signaling has a sporadic success due to the activation of other pathways as well as to the development of chemoresistance. It is known that oxidative stress is closely related to carcinogenesis and to resistance toward classical drug treatment. Therefore, the use of molecules able to reduce STAT3 activation and, on the other hand, to induce a mild oxidative stress in a high-reduced cellular environment may potentially improve cancer treatment outcome. In this context, SLs are promising compounds in cancer drug discovery and their anti-STAT3 activity as well as their ability to disrupt redox homeostasis place them as lead compounds in the development of innovative therapies (Figure 5).

## Figures and Tables

**Table 1:** STAT3-regulated genes.

Tumor-supporting functions of STAT3			
Biological functions	Genes		References
Apoptosis	Bcl-2	↑	[44]
	Mcl-1	↑	[24, 30]
	Bcl-xL	↑	[25, 30]
	Survivin	↑	[26, 30]
	Skp2	↑	[27]
	Fas	↓	[28]

Proliferation	c-Myc	↑	[30]
	Pim-1	↑	[30]
	Cyclin-D1	↑	[23]

Angiogenesis	VEGF	↑	[29, 31]
	bFGF	↑	[32]

Immune suppression	IL-10	↑	[33]
	IL-12	↓	[34]

Invasion and metastasis	MMP-1	↑	[30, 36]
	MMP-2	↑	[30, 35]
	MMP-3	↑	[36]
	MMP-9	↑	[36, 37]
	Vimentin	↑	[38]
	TWIST-1	↑	[39]
	p53	↓	[45]

Cancer stem cell	CPT1B	↑	[40]
Self-renewal	ALDH1A1	↑	[41]
Chemoresistance	SOX2	↑	[42]

**Table 2:** Anti-STAT3 SLs.

Compound	Cell lines/murine model	STAT3 signaling	Molecular mechanism of STAT3 inhibition		Oxidative stress		Biological effect
Alantolactone	A549, NCI-H1650 [139]	p^Tyr705^STAT3 STAT3 DNA binding	↓ ↓	STAT3 glutathionylation	ROS GSH/GSSG	↑ ↓	Apoptosis Enhanced chemosensitivity
	HepG2 [140]	p^Tyr705^STAT3	↓	STAT3 glutathionylation?	ROS [GSH]	↑ ↓	Apoptosis
	MDA-MB231, MCF-7 [141]	p^Tyr705^STAT3	↓	STAT3 glutathionylation?	ROS [GSH]	↑ ↓	Apoptosis
	BxPC-3, AsPC-1, PANC-1 Athymic BALB/cA [142]	p^Tyr705^STAT3	↓	Binding to STAT3 SH2 domain	No evaluated		Cytotoxicity Inhibited cell migration Enhanced chemosensitivity

Santamarine	HepG2 [143]	p^Tyr705^STAT3 pSrc	↓ ↓	STAT3 glutathionylation?	ROS GSH/GSSG	↑ ↓	Apoptosis

Parthenolide	SGC-7901/DDP [144]	p^Tyr705^STAT3	↓	?	No evaluated		Apoptosis Inhibited cell migration and invasion Enhanced chemosensitivity
	HepG2, HT-29, Lovo, MDA-MB-231, MDA-MB-468, HCT116, H460, NCI-H1299, Colo205, BGC [145]	p^Tyr705^STAT3 p^Tyr1007/1008^ JAK2	↓ ↓	Binding to JAK2	ROS	↑	Cytotoxicity

Costunolide	THP1 [126]	p^Tyr705^STAT3 p^Tyr1007/1008^ JAK2 p^Tyr1022/1023^ JAK1 p^Tyr1054/1055^TyK2	↓ ↓ ↓ ↓	STAT3 glutathionylation?	ROS GSH/GSSG	↑ ↓	

1*β*-hydroxyl-5*α*-chloro-8-epi-xanthatin	SK-Hep-1, HepG2, SMMC-7721 [146]	p^Tyr705^STAT3 p^Tyr1007/1008^ JAK2	↓ ↓	STAT3 glutathionylation?	ROS GSH/GSSG	↑ ↓	Cytostatic Apoptosis

Bigelovin	HCT 116 HT-29 26-M01 BALB/c nude mice [147, 148]	p^Tyr705^STAT3 STAT3	↓ ↓	STAT3 glutathionylation?	ROS	↑	Inhibited cell migration and invasion Apoptosis

Dehydrocostuslactone	THP1 [126]	p^Tyr705^STAT3 p^Tyr1007/1008^ JAK2 p^Tyr1022/1023^ JAK1 p^Tyr1054/1055^TyK2	↓ ↓ ↓ ↓	STAT3 glutathionylation?	ROS GSH/GSSG	↑ ↓	
	MCF-7, MDA-MB-231 BALB/cA-nu [149]	p^Tyr1007/1008^ JAK2 p^Tyr1022/1023^ JAK1 p^Tyr705^STAT3	↓ ↓ ↓	SOCS-1 ↑ SOCS-3 ↑	No evaluated		Cell cycle arrest Apoptosis
	K562 [150]	p^Tyr1007/1008^ JAK2 p^Tyr705^STAT3	↓ ↓	?	ROS	↑	Apoptosis

Cynaropicrin	THP-1 DU-145 [127]	p^Tyr705^STAT3 STAT3 DNA binding	↓ ↓	STAT3 glutathionylation?	ROS [GSH]	↑ ↓	Apoptosis Enhanced chemosensitivity

Deoxyelephantopin	HCT 116, K562, KB, T47D [151]	p^Tyr705^STAT3	↓	STAT3 glutathionylation?	ROS	↑	Cytotoxicity Apoptosis Autophagy
	B16-F10, MeWo A375, A2058, SK-MEL-2 NOD/SCID mice [152]	p^Tyr705^STAT3	↓	STAT3 glutathionylation?	ROS	↑	Cytotoxicity Apoptosis Enhanced chemosensitivity

6-O-angeloylplenolin	NCI-H1975, L78, NCI-292, HCC827, A549, 16HBE, BEAS-2B SCID mice [153]	p^Tyr705^STAT3	↓	Binding to STAT3 SH2 domain	Not evaluated		Apoptosis
	MM.1S, MM.1R, U266 BALB/c nude mice [154]	p^Tyr705^STAT3 p^Tyr1007/1008^ JAK2	↓ ↓	?	Not evaluated		Apoptosis Enhanced chemosensitivity

Antrocin	A549, H1975, H441, PC9, BEAS-2B Mice [155]	p^Tyr705^STAT3 p^Tyr1007/1008^ JAK2 p^Tyr1022/1023^ JAK1 p^Tyr1054/1055^TyK2 STAT3 DNA binding	↓ ↓ ↓ ↓ ↓	?			Apoptosis
